# Comparison of the efficacy and safety of perioperative immunochemotherapeutic strategies for locally advanced esophageal cancer: a systematic review and network meta-analysis

**DOI:** 10.3389/fimmu.2024.1478377

**Published:** 2024-12-06

**Authors:** Jiao Zhang, Peixi Zhao, Rui Xu, Le Han, Wenjuan Chen, Yili Zhang

**Affiliations:** ^1^ Department of Pharmacy, Shaanxi Province Tumor Hospital of Xi’an Jiaotong University, Xi’an, China; ^2^ Key Laboratory of Biomedical Information Engineering of Ministry of Education, School of Life Science and Technology, Xi’an Jiaotong University, Xi’an, China; ^3^ Department of Oncology, Shaanxi Province Tumor Hospital of Xi’an Jiaotong University, Xi’an, China; ^4^ Department of Chest Surgery, Shaanxi Province Tumor Hospital of Xi’an Jiaotong University, Xi’an, China

**Keywords:** chemotherapy, immunotherapy, neoadjuvant therapy, esophageal carcinoma, meta-analysis

## Abstract

**Background:**

The aim of this network meta-analysis was to clarify the efficacy and safety of different immune checkpoint inhibitors (ICIs) in combination with chemotherapy in the neoadjuvant phase for the treatment of locally advanced esophageal cancer.

**Methods:**

We searched PubMed, EMBASE, Web of Science, Cochrane Library, CNKI and WanFang databases from January 2000 until May 2024. The primary endpoints were pathological complete response (pCR), major pathological response (MPR), R0 resection rate, objective response rate (ORR), disease control rate (DCR), treatment-related adverse events(TRAEs) of any grade and TRAEs of grade 3 or higher. The Newcastle-Ottawa Scale (NOS) and the Cochrane Risk of Bias tool were used to evaluate risk of bias. To analyze the data, Review Manager 5.3 and Stata16.0 were applied.

**Results:**

Fourteen eligible studies (six randomized controlled trials) and 8 retrospective cohort studies) enrolling 1139 patients were included for this network meta-analysis. All studies originated from China. For patients with locally advanced esophageal cancer, neoadjuvant immunochemotherapeutic strategies showed significant advantages over traditional neoadjuvant therapy in terms of pCR, MPR, ORR and DCR. Among the analyzed regimens, camrelizumab plus chemotherapy demonstrated the most pronounced improvements in pCR and MPR, while pembrolizumab plus chemotherapy achieved the best outcomes in terms of ORR and DCR. There were no significant differences observed among the various neoadjuvant treatment strategies regarding R0 resection rate, any grade TRAEs, or grade≥3 TRAEs. The most common TRAEs in the neoadjuvant chemotherapy plus immunotherapy group were myelosuppression and gastrointestinal damage, with most grade 3 or higher TRAEs being hematologic adverse events. The most frequent immune-related adverse events(irAEs) included rash (4.2-21.7%), thyroid dysfunction (hypothyroidism or hyperthyroidism, 6.3-17.4%), and pneumonia (4.2-6.3%), with the majority being mild to moderate (grade 1 or 2).

**Conclusions:**

Neoadjuvant immunotherapy combined with chemotherapy regimens demonstrate relatively high efficacy and tolerable safety profiles. Among the evaluated regimens, the combination chemotherapy with camrelizumab had relatively high pCR and MPR, whereas the combination chemotherapy with pembrolizumab had relatively high ORR and DCR. There were no significant differences in safety among the various regimens. Our study suggests that evaluating the efficacy and safety of different ICIs may be helpful in clinical decision-making.

**Systematic review registration:**

https://www.crd.york.ac.uk/prospero/, identifier CRD42024583548.

## 1 Introduction

Esophageal cancer is a tumor of the digestive system with high malignancy, high morbidity, and high mortality. According to the latest statistics from the International Agency for Research on Cancer, esophageal cancer is the 11th most commonly diagnosed cancer and the seventh leading cause of cancer death worldwide, with an estimated 511,000 new cases and 445,000 deaths in 2022 (1). In China, there were 224,000 new cases of esophageal cancer in 2022, ranking 7th in incidence, with 187,500 deaths, ranking 5th in mortality (2). The two primary histologic subtypes of esophageal cancer are esophageal adenocarcinoma (EAC) and esophageal squamous cell carcinoma (ESCC). EAC is the predominant subtype in patients with esophageal cancer in the United States and Europe, accounting for approximately 70% of cases, while in Asia, 90% or more esophageal cancers are ESCC (3). Some data show that more than half of esophageal cancer patients are in locally advanced or advanced stages at the time of diagnosis. The treatment outcome of esophageal cancer is usually poor, the five-year survival rate for locally advanced esophageal cancer is 46.7%, while for esophageal cancer with distant metastasis, the five-year survival rate is only 4.8%.Therefore, esophageal cancer deserves our attention since it can be a serious threat to human life and health.

At present, the guidelines of National Cancer Comprehensive Network and Chinese Society of Clinical Oncology both suggest that neoadjuvant chemoradiotherapy (nCRT) and neoadjuvant chemotherapy (nCT) should be the standard treatment modes for patients with locally advanced esophageal cancer (4, 5). According to the long-term follow-up results of the CROSS and NEOCRTEC5010 trials, nCRT combined with surgery significantly improved the overall survival and disease-free survival among patients with locally advanced esophageal cancer (6, 7). According to the results of NEOCRTEC5010 trials, patients receiving nCRT plus surgery had prolonged overall survival compared with those receiving surgery alone (hazard ratio, 0.74; 95% CI, 0.57-0.97; P = 0.03), with a 5-year survival rate of 59.9% (95% CI,52.9%-66.1%) vs 49.1% (95% CI, 42.3%-55.6%), respectively. The results of two multicenter, prospective, randomized phase III clinical trials (JCOG9907 and JCOG1109) suggest that preoperative neoadjuvant chemotherapy significantly improves long-term survival in patients with resectable locally advanced ESCC (8, 9). However, after neoadjuvant therapy, the postoperative recurrence and metastasis rates of locally advanced esophageal cancer are still high, and the long-term survival rate is relatively low. Therefore, the pursuit of more enhanced neoadjuvant therapeutic models is of crucial significance in further augmenting the prognosis of patients with locally advanced esophageal cancer, mitigating adverse reactions, and minimizing the disruptive impact on surgical interventions.

Immune therapy induces tumor cell lysis and apoptosis by stimulating the body’s immune cells, reducing or disrupting the tumor’s immunosuppressive microenvironment, thereby restoring the patient’s anti-tumor immune response (10). Immune checkpoint inhibitors (ICIs) have been proven to be effective in the treatment of various types of cancer, including as first-line treatment for advanced esophageal cancer (11–14). The research results of KEYNOTE-590 indicated that pembrolizumab combined with chemotherapy performed better than placebo combined with chemotherapy in terms of overall survival in patients with ESCC (12.6 months vs 9.8 months; 0.72 (0.60-0.88); p=0.0006) (15). Furthermore, strategies combining radiochemotherapy or chemotherapy with ICIs have been used in neoadjuvant therapy for resectable locally advanced esophageal cancer, showing promising results (16–18). Some clinical trials suggest that combining ICIs with chemotherapy in neoadjuvant therapy can induce a pCR rate of 16.7-45% in esophageal cancer.

Currently, traditional meta-analyses have compared the safety and efficacy of neoadjuvant immune therapy versus traditional chemotherapy in esophageal cancer. However, no studies have yet determined the optimal immune checkpoint inhibitor for neoadjuvant immune combination therapy in esophageal cancer. Therefore, this study employs network meta-analysis to explore the safety and efficacy of different combinations of ICIs with chemotherapy as neoadjuvant treatment strategies for esophageal cancer, aiming to provide clinical decision-making guidance.

## 2 Methods

### 2.1 Literature search strategy

A comprehensive search of the literature was conducted using the following databases: PubMed, EMBASE, Web of Science, the Cochrane Library, China National Knowledge Infrastructure (CNKI), and WanFang. The search was limited to studies published from January 2000 until May 2024.The search keywords were “nivolumab OR pembrolizumab OR camrelizumab OR sintilimab OR toripalimab OR tislelizumab OR atezolizumab OR durvalumab OR avelumab OR ipilimumab OR tremelimumab OR lambrolizumab OR cemiplimab OR programmed cell death 1 (PD-1) OR programmed cell death ligand 1 (PD-L1) OR cytotoxic T-lymphocyte-associated antigen 4 (CTLA-4) OR immunotherapy OR immune checkpoint inhibitors”, “neoadjuvant OR preoperative OR perioperative”、”esophageal OR esophagus OR oesophageal OR oesophagus”. The search query is provided in **Supplementary Table 1**. Manual searches of the references in the systematic reviews and included studies were conducted to identify any additional qualified research.

### 2.2 Inclusion criteria

Studies that meet the following criteria are included in the meta-analysis: 1) Study design: randomized controlled trials (RCTs) or cohort studies; 2) Participants: Patients with resectable esophageal cancer confirmed by histological examination, regardless of their region, age, or race; 3) Interventions: The studies comparing neoadjuvant ICIs in combination with chemotherapy and neoadjuvant chemotherapy alone were included in this analysis; 4) Outcomes: pathological complete response (pCR), major pathological response (MPR), R0 resection rate, objective response rate (ORR), disease control rate (DCR), treatment-related adverse events(TRAEs) of any grade and TRAEs of grade 3 or higher.

### 2.3 Exclusion criteria

Studies meeting the following criteria were excluded: 1) Patients who had received anti-esophageal cancer treatment prior to neoadjuvant therapy; 2) Studies unable to provide at least one of the aforementioned outcome measures; 3) Studies published as case reports, reviews, or expert opinions; 4) Studies with significant baseline imbalances among patients.

### 2.4 Data extraction and outcomes measures

Two investigators (JZ and RX) conducted the literature retrieval, data extraction, and quality assessment in an independent manner. The following information from included studies was extracted: 1) Study characteristics, including first author, year of publication, country, study design, sample size, details regarding interventions and outcomes; 2) Baseline characteristics of enrolled patients, such as gender, age, histological type of tumor, clinical TNM staging of tumors; 3) Endpoint data, including pCR, MPR, R0 resection rate, ORR, DCR, incidence of TRAEs of any grade and TRAEs of grade≥3.

### 2.5 Quality of evidence and risk of bias

Two researchers independently evaluated the quality of the included cohort studies using the Newcastle Ottawa Scale (NOS) (19). Studies with an NOS score ≥ 6 were considered high quality; otherwise, they were deemed low quality. The quality of RCTs was evaluated using the Cochrane Risk of Bias Assessment Tool (20). This tool evaluates random sequence generation, allocation concealment, blinding of participants and personnel to group allocation, blinding of outcome assessment, completeness of outcome data, presence of reporting bias, and other potential biases. These assessments involve seven items across six domains. Studies were categorized as “low risk of bias”, “unclear risk of bias”, or “high risk of bias” based on these criteria.

### 2.6 Statistical analysis

Data analysis was performed using STATA 16.0 software. Multivariate network meta-analysis (NMA) was conducted within a frequency statistics framework using the “network” command in STATA. To visualize the results of NMA, interval plots were generated using the ‘intervalplot’ command in STATA. Interval plots were used to summarize and display the confidence intervals (CIs) and matching prediction intervals (Prls) for all comparisons. For each outcome, we estimated the relative ranking of the different therapeutic regimens according to the distribution of the surface under the cumulative ranking (SUCRA) probabilities. Increased SUCRA values indicated higher efficacy and probability of adverse events. We use the standardized mean differences (SMDs) with 95% confidence intervals(CIs) for consecutive outcomes and the odds ratio (OR) with 95% CIs for dichotomous outcomes as effect measures.

## 3 Results

### 3.1 Search results and study characteristics

**Figure 1:**
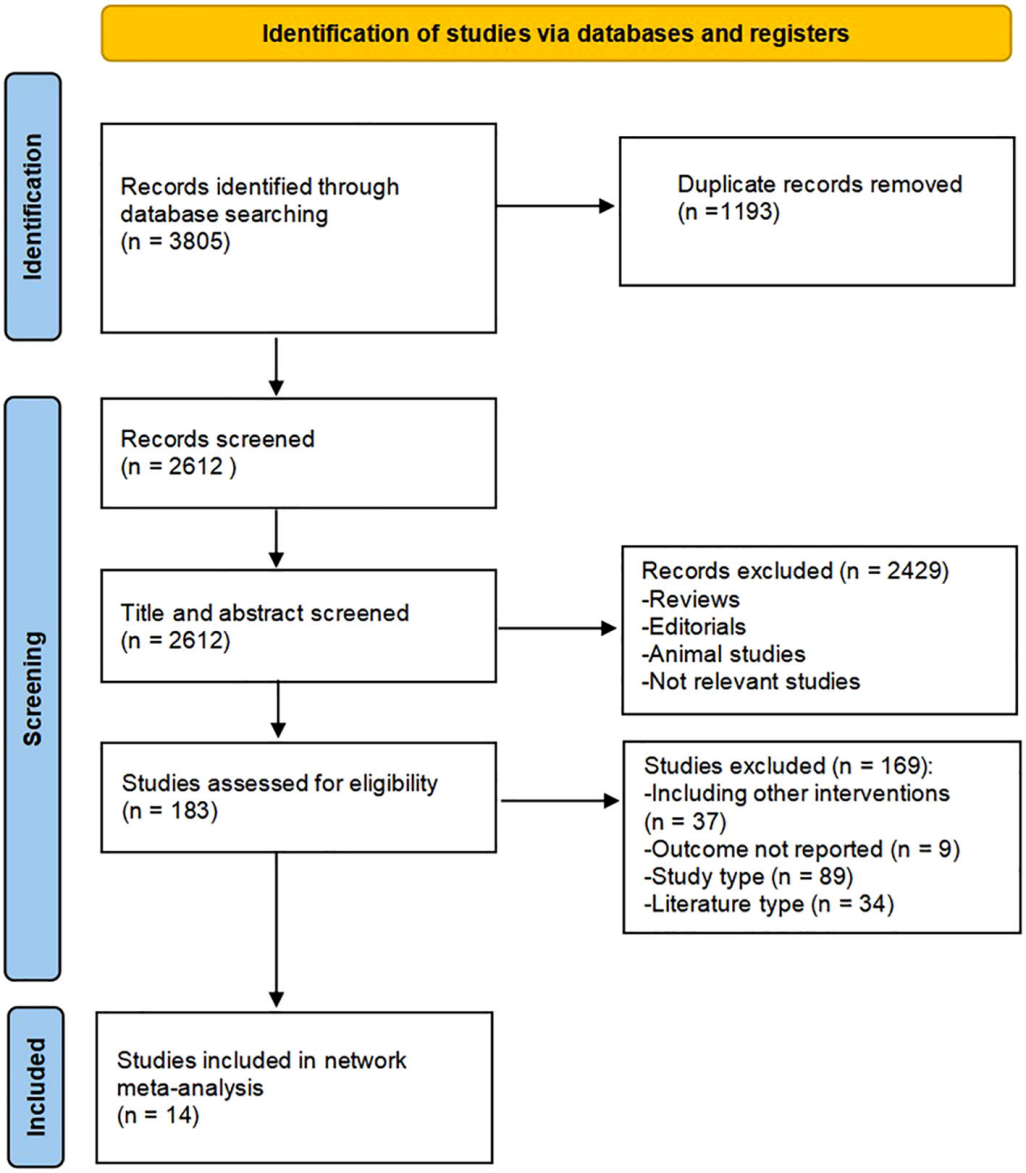
Flow diagram of included studies for this meta-analysis.

A detailed overview of PRISMA flow chart for database searching and study identification is presented in **Figure 1**. Fourteen studies enrolling 1139 participants were included in this meta-analysis. Characteristics of included studies and research data on endpoints were summarized in **Tables 1** and **2**, respectively.

**Table 1:** Characteristics of the included studies.

First author/ Year	Country	Study design	Histological type	Clinical stage	Intervention	ICI(dose)	CT regimen	Cycles Of nICT	The duration of one treatment cycle	Sample size	Age(years)	Gender males, n (%)
Yong Li 2023 (21)	China	Prospective	ESCC	II-IVa	nICT	Socazolimab	nab-paclitaxel+ cisplatin	4	NR	32	61 (53–72)	23(71.9)
					nCT					32	63(47–74)	28(87.5)
Yong Xiao 2021 (22)	China	Prospective	ESCC	II-III	nICT	Camrelizumab (200mg)	docetaxel+oxaliplatin	4	2w	30	42.67± 15.57	15(50)
					nCT					30	43.61 ± 12.54	14(46.7)
Yujin Qiao 2022 (23)	China	Retrospective	ESCC	I;-IVa	nICT	Camrelizumab (200mg)	paclitaxel/nab-paclitaxel/docetaxel+platinum	2	3w	48	64.15 ± 7.29	38(79.2)
					nCT					206	62.22 ± 7.14	147(71.4)
Shu Wang 2023 (24)	China	Prospective	ESCC	II-IVa	nICT	Camrelizumab (200mg)	docetaxel+cisplatin+ fluorouracil	3	3w	15	61 ± 7.8	13(86.7)
					nCT					15	63 ± 7.6	15(100)
RuiqinZhou 2023 (25)	China	Retrospective	ESCC	II-IVa	nICT	Camrelizumab (200mg)	docetaxel+nedaplatin	2	3w	19	65.89 ± 6.06	17(89.5)
					nCT					40	64.50 ± 4.54	31(77.5)
Renquan Zhang 2023 (26)	China	Prospective	ESCC	cT1-4N1-3M0、 cT3-4N0M0	nICT	Camrelizumab (200mg)	nab-paclitaxel+ cisplatin	NR	NR	90	65	NR
					nCT					60	65	NR
Baihua Zhang 2023 (27)	China	Retrospective	ESCC	T2-4N+	nICT	Camrelizumab (200mg)	paclitaxel+platinum	1-4	3w	34	60.68 ± 7.44	31(91.2)
					nCT					97	60.08 ± 7.78	31(91.2)
XiaolinLi 2023 (28)	China	Retrospective	ESCC	III-IV	nICT	Camrelizumab (200mg)	nab-paclitaxel+ nedaplatin	2-3	3w	30	61.4 ± 7.87	20(66.7)
					nCT					30	63.4 ± 6.33	21(70)
Xianfang Chen 2023 (29)	China	Prospective	esophageal cancer	IIIb-IV	nICT	Camrelizumab (200mg)	nab-paclitaxel+ cisplatin	2	3w	37	61.10 ± 3.26	30(81.1)
					nCT					36	60.22 ± 3.45	29(80.6)
Bingjiang Huang 2021 (30)	China	Retrospective	ESCC	II-IVa	nICT	pembrolizumab (200mg)	docetaxel+ nidaplatin	2	3w	23	59.2 ± 7.3	21(91.3)
					nCT					31	58.9 ± 6.4	30(96.7)
XinweiZhang 2022 (31)	China	Prospective	esophageal cancer	II-III	nICT	pembrolizumab (200mg)	nab-paclitaxel+ nedaplatin	NR	3w	30	57.91 ± 8.06	30(100)
					nCT					29	56.7 ± 7.95	29(100)
Xuezhong Wang 2023 (32)	China	Retrospective	ESCC	IIa-III	nICT	pembrolizumab (200mg)	pemetrexed+ cisplatin	2	3w	57	57.13 ± 9.11	33(57.9)
					nCT					58	58.80 ± 9.21	36(62.1)
Jing Chen 2021 (33)	China	Prospective	ESCC	II-III	nICT	pembrolizumab (200mg)	pemetrexed+ cisplatin	2	3w	49	56.37 ± 5. 81	31(63.3)
					nCT					49	54.86 ± 7.05	35(71.4)
Chunlin Li 2023 (34)	China	Retrospective	esophageal cancer	III	nICT	Sintilimab (200mg)	nab-paclitaxel+ nedaplatin	2	3w	40	65.3 ± 5.84	27(67.5)
					nCT					42	65.5 ± 6. 11	28(66.7)

ESCC, esophageal squamous cell cancer; ICI, immune checkpoint inhibitor; nCT, neoadjuvant chemotherapy; nICT, neoadjuvant immune checkpoint inhibitors combined with chemotherapy; NR, not reported.

**Table 2:** Research data on endpoints of included studies.

First author/ Year	Treatment	pCR rate(%)	MPR rate(%)	R0 resection rate(%)	ORR (%)	DCR (%)	Incidence of TRAEs, (%)	Incidence of grage ≥ 3 TRAEs, (%)	Neutropenia(%)	Leukopenia(%)	Anaemia(%)	Thrombocytopenia(%)	Gastrointestinal react(%)	Diarrhea(%)	Hypothyroidism or hyperthyroidism(%)	Rash(%)	RCCEP(%)	Pneumonia(%)
Yong Li 2023 (21)	nICT	41.4	69	100	NR	NR	100	65.6	78.1	78.1	100	100	NR	18.8	6.3	12.5	NR	6.3
	nCT	27.6	62	96.6	NR	NR	100	62.5	78.1	68.8	84.4	84.4	NR	6.3	0	0	NR	6.3
Yong Xiao 2021 (22)	nICT	NR	NR	100	NR	NR	13.3	NR	NR	6.7	NR	0	NR	0	NR	NR	NR	NR
	nCT	NR	NR	100	NR	NR	20	NR	NR	10	NR	3	NR	3.3	NR	NR	NR	NR
Yujin Qiao 2022 (23)	nICT	41.7	60	100	NR	NR	77.1	NR	NR	NR	NR	NR	12.5	NR	NR	4.2	54.2	NR
	nCT	10.7	27	100	NR	NR	91.7	NR	NR	NR	NR	NR	9.2	NR	NR	0.5	0	NR
Shu Wang 2023 (24)	nICT	NR	NR	NR	46.7	93.3	NR	33.3	26.7	40	53.3	36.7	NR	6.7	NR	NR	NR	NR
	nCT	NR	NR	NR	26.7	86.7	NR	13.4	6.7	13.3	26.7	20	NR	6.7	NR	NR	NR	NR
RuiqinZhou 2023 (25)	nICT	26.3	NR	100	NR	NR	84	5.3	5.3	31.6	10.5	5.3	NR	0	10.5	NR	21.1	NR
	nCT	2.5	NR	97.5	NR	NR	87.5	7.5	7.5	35	12.5	7.5	NR	5	2.5	NR	0	NR
Renquan Zhang 2023 (26)	nICT	27.8	43.3	NR	NR	NR	NR	NR	NR	NR	NR	NR	NR	NR	NR	NR	NR	NR
	nCT	10	26.7	NR	NR	NR	NR	NR	NR	NR	NR	NR	NR	NR	NR	NR	NR	NR
Baihua Zhang 2023 (27)	nICT	23.5	52.9	NR	79.4	NR	47.1	11.8	NR	NR	NR	NR	2.9	NR	NR	20.6	NR	NR
	nCT	3	16.5	NR	66	NR	38.1	6.2	NR	NR	NR	NR	12.4	NR	NR	1	NR	NR
XiaolinLi 2023 (28)	nICT	40	66.7	100	80	100	NR	NR	NR	NR	NR	NR	43.3	NR	NR	NR	60	NR
	nCT	6.7	30	93.3	43.3	90	NR	NR	NR	NR	NR	NR	50	NR	NR	NR	0	NR
Xianfang Chen 2023 (29)	nICT	NR	NR	NR	43.2	89.2	32.4	0	NR	NR	NR	NR	8.1	8.1	NR	NR	NR	NR
	nCT	NR	NR	NR	25	69.4	55.6	11.1	NR	NR	NR	NR	16.7	13.8	NR	NR	NR	NR
Bingjiang Huang2021 (30)	nICT	30.4	47.8	100	87	95.7	NR	34.8	78.3	82.6	82.6	NR	NR	NR	17.4	21.7	NR	4.3
	nCT	9.7	25.8	96.3	54.8	87.1	NR	25.8	54.8	61.3	58	NR	NR	NR	0	0	NR	0
XinweiZhang 2022 (31)	nICT	NR	NR	NR	78.3	95.7	NR	0	26.1	NR	NR	39.1	NR	NR	NR	NR	NR	NR
	nCT	NR	NR	NR	47.8	82.6	NR	0	34.8	NR	NR	45.7	NR	NR	NR	NR	NR	NR
Xuezhong Wang 2023 (32)	nICT	NR	NR	NR	64.9	89.5	40.4	NR	NR	7	NR	5.3	19.3	NR	NR	NR	NR	NR
	nCT	NR	NR	NR	43.1	86.2	39.7	NR	NR	8.6	NR	3.4	17.2	NR	NR	NR	NR	NR
Jing Chen 2021 (33)	nICT	NR	NR	NR	61.2	91.8	NR	26.5	NR	38.8	NR	24.5	NR	NR	NR	NR	NR	NR
	nCT	NR	NR	NR	40.8	51	NR	36.7	NR	44.9	NR	30.6	NR	NR	NR	NR	NR	NR
Chunlin Li 2023 (34)	nICT	15	50	97.5	70	90	62.5	NR	NR	NR	NR	NR	12.5	NR	12.5	NR	NR	NR
	nCT	4.8	26.2	92.9	40	71.4	66.7	NR	NR	NR	NR	NR	14.3	NR	7.1	NR	NR	NR

pCR, pathological complete response; MPR, major pathological response; ORR, objective response rate; DCR, disease control rate; TRAE, treatment-related adverse event; RCCEP, Reactive Cutaneous Capillary Endothelial Proliferation; nCT,neoadjuvant chemotherapy; nICT, neoadjuvant immune checkpoint inhibitors combined with chemotherapy; NR, not reported.

Seven studies were published in English, while the other seven were published in Chinese with English abstracts. All studies originated from China. Of the included studies, six were RCTs (21, 22, 24, 26, 29, 31), and eight were retrospective cohort studies (23, 25, 27, 28, 30, 32–34). The studies collectively enrolled 1139 patients with esophageal cancer, including 11 studies focused on ESCC and 3 studies on esophageal cancer without distinguishing between ESCC and EAC. The included neoadjuvant therapies consisted of nCT, nCT + Socazolimab, nCT + Camrelizumab, nCT + Pembrolizumab, and nCT + Sintilimab. The primary neoadjuvant chemotherapy regimens used included platinum-based doublet chemotherapy regimens with paclitaxel, albumin-bound paclitaxel, or docetaxel. Two studies used a regimen of pemetrexed + cisplatin, and only one study used docetaxel + cisplatin + 5-fluorouracil.The largest sample size included was 354 cases, and the smallest was 30 cases. The included patients were predominantly male, with a median age exceeding 60 years.

### 3.2 Study quality

In terms of research quality, one study was deemed to be of high risk of bias for inadequate blinding of investigators(open-label). The other studies were assessed to be of some concerns. all the NOS scores of the cohort studies were greater than 6, indicating high quality. The details of quality assessment for included studies are listed in **Supplementary Tables 2** and **3**.

### 3.3 Outcome measures

#### 3.3.1 Pathological complete response rate

**Figure 2:**
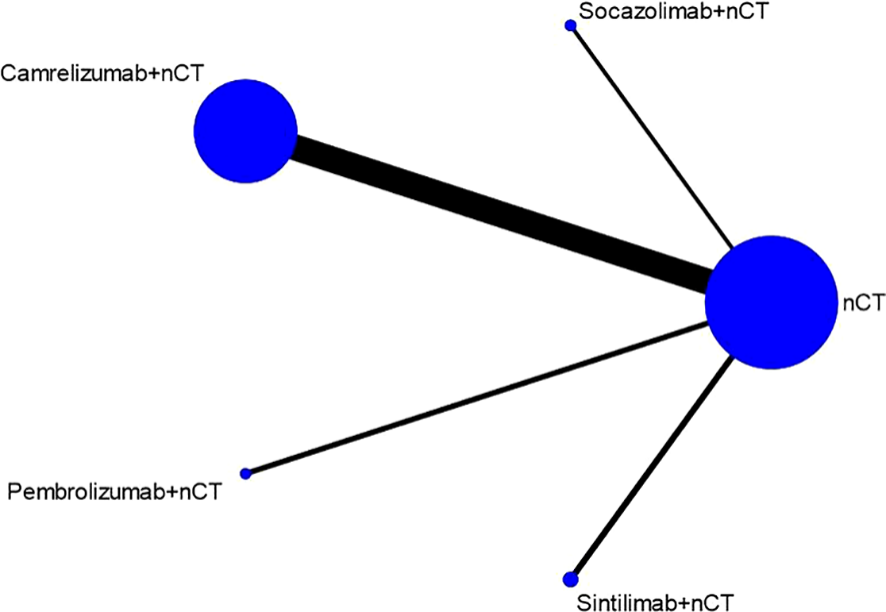
Comparative network plots for pathological complete response (pCR).

Seven studies reported the pCR rate. **Figure 2** shows the evidence network diagram for pCR rate. As illustrated in the figure, each dot represents a distinct intervention measure. The size of each dot is indicative of the number of studies in which this intervention was used. The lines connecting the dots represent direct comparative studies between two intervention measures. The thickness of the lines represents the number of studies conducted for each comparison. The comparison with camrelizumab + nCT versus nCT had the highest number of studies, as the figure shows.

**Figure 3:**
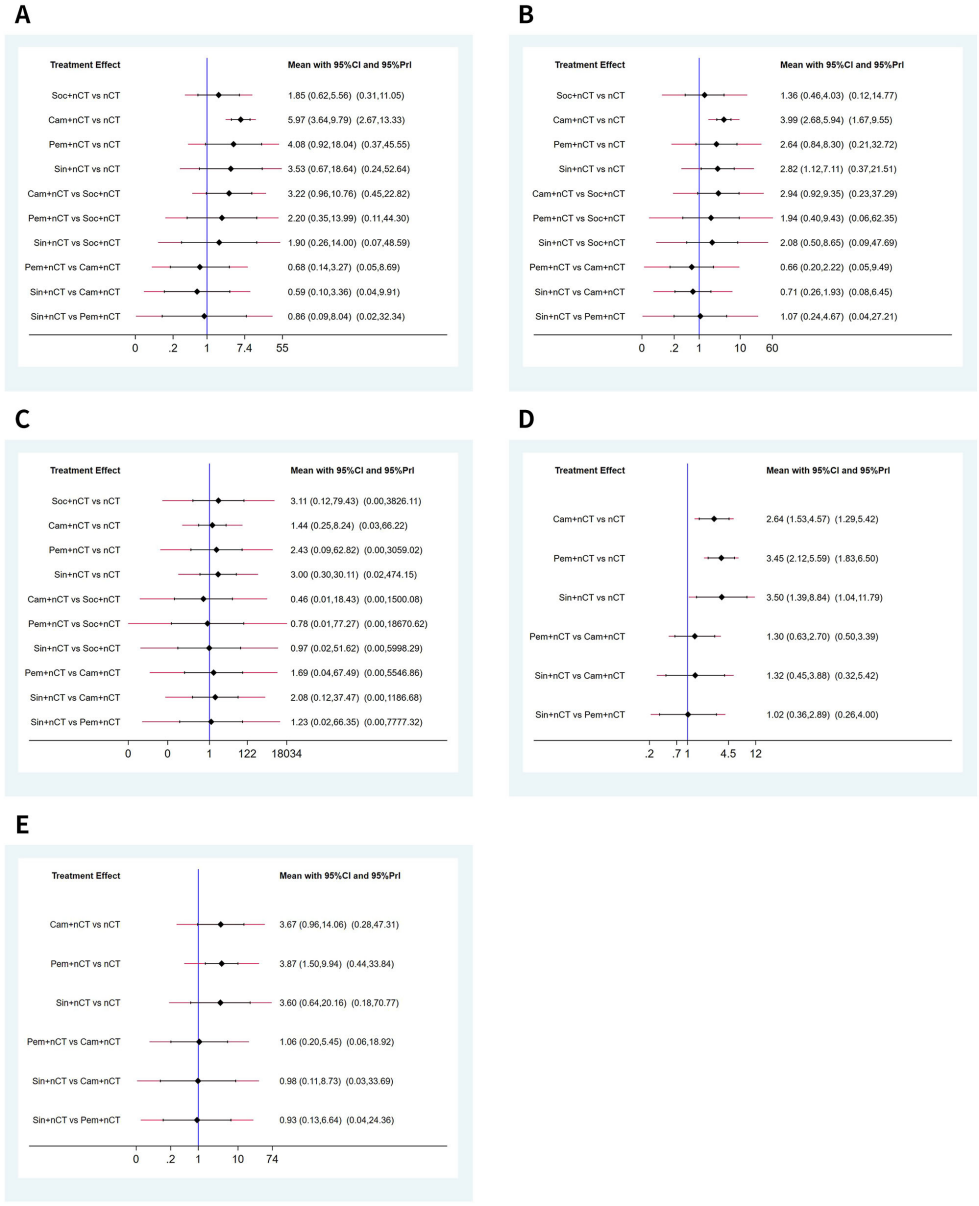
Predictive intervals plots for efficacy of immunochemotherapeutic strategies. **(A)** Pathological complete response (pCR), **(B)** major pathological response (MPR), **(C)** R0 resection rate, **(D)** objective response rate (ORR), **(E)** disease control rate (DCR). The graph presents the network estimates for all pairwise comparisons. Black horizontal lines represent the confidence intervals and red lines represent the predictive intervals.

**Figure 4:**
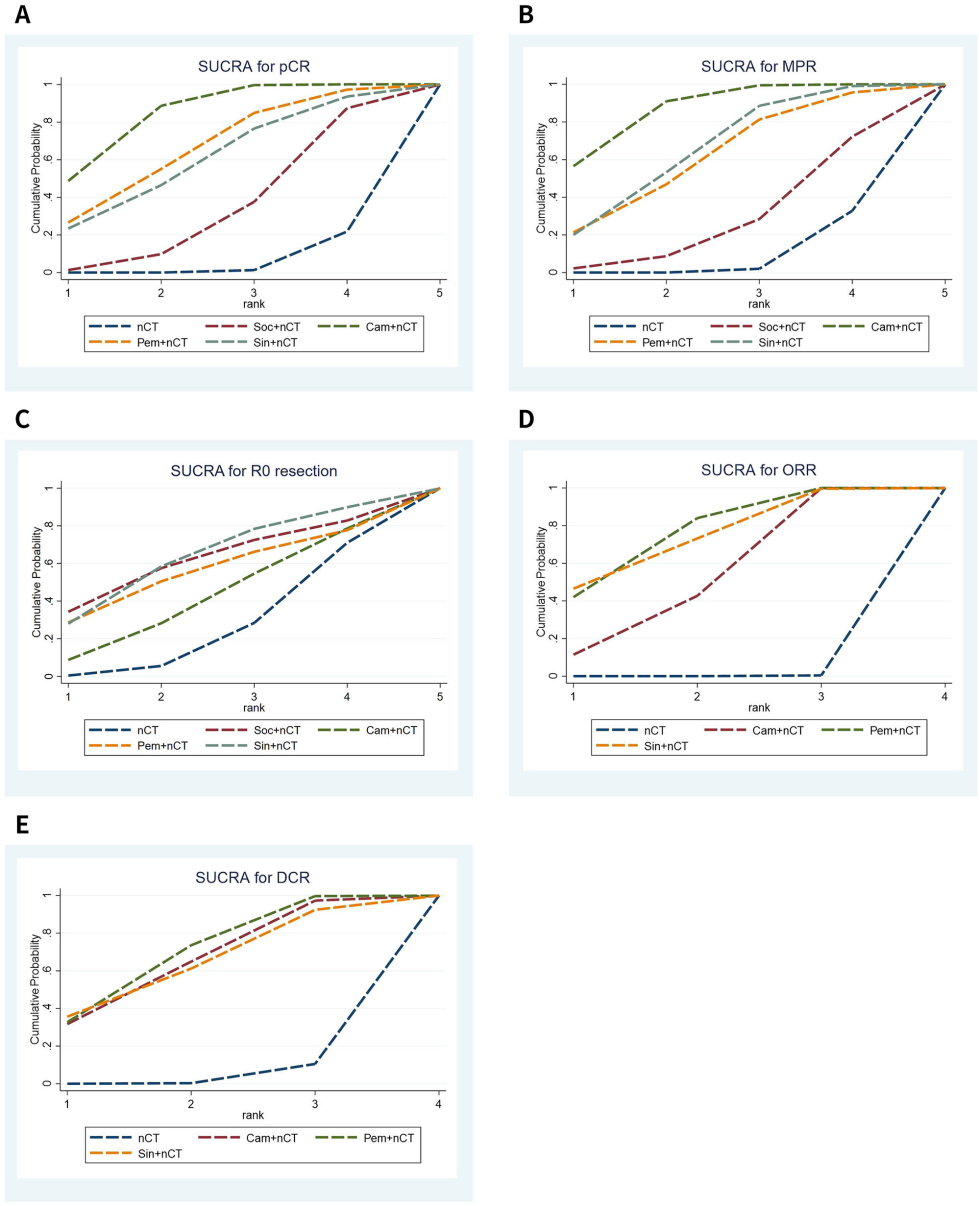
Cumulative ranking probability graphs for efficacy of immunochemotherapeutic strategies. **(A)** Pathological complete response (pCR), **(B)** major pathological response (MPR), **(C)** R0 resection rate, **(D)** objective response rate (ORR), **(E)** disease control rate (DCR).

The NMA results demonstrated that patients receiving neoadjuvant camrelizumab + chemotherapy had a significantly higher postoperative pCR rate (odds ratio 5.97, 95% confidence interval 3.64-9.79) compared to those receiving neoadjuvant chemotherapy alone (**Figure 3A**). The size of SUCRA was directly correlated with the cumulative ranking probability of the intervention method in the cumulative ranking probability graph of the intervention methods. According to the cumulative ranking probability plot (**Figure 4A**), the five neoadjuvant immunotherapy regimens arranged in descending order based on their postoperative pCR rates were camrelizumab + chemotherapy (probability, 84.2%), pembrolizumab + chemotherapy (probability, 66.0%), sintilimab + chemotherapy (probability, 60.0%), socazolimab + chemotherapy(probability, 34.0%), nCT (probability, 5.8%).

#### 3.3.2 Major pathological response rate

Seven studies reported comparison of MPR rate, **Supplementary Figure 1A** shows the evidence network diagram of MPR rate. NMA results displayed that patients who received neoadjuvant camrelizumab + chemotherapy (odds ratio 3.99, 95% confidence interval 2.68-5.94), sintilimab + chemotherapy (odds ratio 2.82, 95% confidence interval 1.12-7.11) had substantially higher postoperative MPR rates than those who received neoadjuvant chemotherapy alone (**Figure 3B**). The cumulative ranking probability plot illustrated that camrelizumab + chemotherapy has the highest MPR rate (probability, 86.7%), followed by sintilimab + chemotherapy (probability, 65.5%), pembrolizumab + chemotherapy (probability, 61.3%) and socazolimab + chemotherapy (probability, 27.6%), and nCT group has the lowest MPR rate(probability, 8.9%), as plotted in **Figure 4B**.

#### 3.3.3 R0 resection rate

Seven studies reported comparison of R0 resection rate, **Supplementary Figure 1B** shows the evidence network diagram of R0 resection rate. NMA results displayed no significant difference among all treatment modalities, as shown in (**Figure 3C**). The cumulative ranking probability plot illustrated that sintilimab + chemotherapy has the highest R0 resection rate (probability, 63.7%), followed by socazolimab + chemotherapy (probability, 61.8%), pembrolizumab + chemotherapy (probability, 55.7%) and camrelizumab + chemotherapy (probability, 42.5%) has the lowest R0 resection rate (probability, 26.3%), as plotted in **Figure 4C**.

#### 3.3.4 Objective response rate

Nine studies involving four interventions reported the results of ORR, and **Supplementary Figure 1C** shows the evidence network diagram of ORR. As shown in **Figure 3D**, compared to neoadjuvant chemotherapy alone, neoadjuvant immunotherapy combined with chemotherapy significantly improves ORR. Among these, sintilimab + nCT showed the highest improvement in ORR compared to nCT alone (odds ratio 3.50, 95% confidence interval 1.39-8.84), followed by pembrolizumab + nCT (odds ratio 2.64, 95% confidence interval 1.53-4.57) and camrelizumab + nCT (odds ratio 2.82, 95% confidence interval 1.12-7.11). According to the cumulative ranking probability plot, the postoperative ORR in descending order for the four neoadjuvant immunotherapy regimens were: pembrolizumab + chemotherapy (probability, 75.3%), sintilimab + chemotherapy (probability, 73.1%), camrelizumab + chemotherapy (probability, 51.4%), nCT (probability, 0.1%), as plotted in **Figure 4D**.

#### 3.3.5 Disease control rate

Eight studies involving four interventions reported the results of DCR, and **Supplementary Figure 1D** shows the evidence network diagram of DCR. As shown in **Figure 3E**, compared to neoadjuvant chemotherapy alone, pembrolizumab + chemotherapy significantly improves DCR (odds ratio 3.87, 95% confidence interval 1.50-9.94). According to the cumulative ranking probability plot, the postoperative DCR in descending order for the four neoadjuvant immunotherapy regimens were: pembrolizumab + chemotherapy (probability, 68.7%), camrelizumab + chemotherapy (probability, 64.7%), sintilimab + chemotherapy (probability, 63.0%), nCT (probability, 3.6%).as plotted in **Figure 4E**.

#### 3.3.6 Safety analysis

**Supplementary Figures 1E, F** respectively depict the network evidence graphs for TRAEs of any grade and TRAEs of grade 3 or higher. In terms of safety analysis, the NMA results indicate no significant differences observed among the interventions in terms of TRAEs of any grade or grade≥3(**Supplementary Figure 2A**; **Supplementary Figure 2B**). The five perioperative immunochemotherapy regimens arranged in descending order based on TRAEs of any grade were: nCT (probability, 62.8%), pembrolizumab + chemotherapy(probability, 59.3%), socazolimab + chemotherapy (probability, 53.3%), sintilimab + chemotherapy (probability, 48.7%), camrelizumab + chemotherapy (probability, 25.9%) (**Supplementary Figure 3A**).The four perioperative immunochemotherapy regimens arranged in descending order based on TRAEs of grade≥3 were: camrelizumab + chemotherapy (probability, 67.0%), socazolimab + chemotherapy(probability, 58.2%), nCT (probability, 45.4%), pembrolizumab + chemotherapy(probability, 29.4%), as plotted in **Supplementary Figure 3B**.

## 4 Discussion

Neoadjuvant synchronous chemoradiotherapy or neoadjuvant chemotherapy followed by surgery remains the primary treatment choice for locally advanced esophageal cancer. Recently, combining immunotherapy with chemotherapy during the neoadjuvant phase has demonstrated impressive efficacy and safety in treating esophageal cancer. Preclinical studies indicate that PD-1 inhibitors combined with chemotherapy can further enhance host immune responses and suppress immune escape by cancer cells (35). To enhance efficacy, neoadjuvant immunotherapy is often combined with chemotherapy or chemoradiotherapy (36). However, it remains unclear which immune checkpoint inhibitor, when combined with chemotherapy or chemoradiotherapy, offers the best efficacy and highest safety. To our knowledge, this is the first network meta-analysis evaluating the safety and efficacy of different ICIs combined with chemotherapy regimens as neoadjuvant treatment strategies for esophageal cancer.

The results of this study indicate that perioperative camrelizumab plus chemotherapy confer greater benefits in terms of postoperative pathological response compared to traditional neoadjuvant chemotherapy and other ICIs plus chemotherapy in esophageal cancer. Camrelizumab plus chemotherapy correlates with optimal rates of pCR and MPR, consistent with some previous studies. Traditional meta-analyses comparing the efficacy and safety of neoadjuvant immunotherapy combined with chemoradiotherapy or chemotherapy versus standard neoadjuvant strategies (nCT and nCRT) have shown that neoadjuvant immunotherapy (nICT and nICRT) significantly improves pCR and MPR rates in locally advanced esophageal cancer (16, 37–39). Additionally, Mei et al. elucidated the efficacy and safety of perioperative immunotherapy strategies for resectable non-small cell lung cancer, suggesting no significant difference in pCR benefits between camrelizumab plus chemotherapy and pembrolizumab plus chemotherapy, but camrelizumab plus chemotherapy ranked higher than pembrolizumab plus chemotherapy and chemotherapy in terms of probability (40). Furthermore, achieving R0 resection is a key criterion for assessing the effectiveness of surgical interventions in esophageal cancer because it is associated with improved patient prognosis and serves as a benchmark for successful treatment outcomes. In this study, there were no significant differences in the R0 resection rate among the intervention measures, and all achieved high R0 resection rate. These results suggest that neoadjuvant immunotherapy combined with chemotherapy strategies are effective in achieving complete resection with negative margins for tumors.

Short-term efficacy in tumor treatment refers to how well a cancer therapy works in the initial phase of treatment, typically evaluated within weeks to a few months. It assesses the immediate effects of a treatment on tumor size, growth rate, and symptom relief, often serving as a measure for determining whether the treatment approach is worth continuing. In terms of short-term efficacy, this study showed that pembrolizumab + nCT had the best ORR benefit, followed by sintilimab + chemotherapy, camrelizumab + nCT, and nCT had the lowest ORR improvement. Li et al. (41) clarified the safety and efficacy of various ICIs plus chemotherapy in advanced esophageal cancer, and compared with chemotherapy alone, immunotherapy combined with chemotherapy improved the ORR, moreover, in terms of ORR benefit, pembrolizumab + chemotherapy was the best, followed by sintilimab + chemotherapy, and camrelizumab + chemotherapy had the lowest ORR benefit, which was consistent with the findings of this study. In addition, in a comparative analysis of the efficacy and safety of immunotherapy for patients with advanced or metastatic ESCC by Gao et al. (42), it was concluded that there was no statistical difference in terms of ORR benefit between first-line and second-line treatments for advanced esophageal cancer with respect to pembrolizumab + chemotherapy, sintilimab + chemotherapy, and camrelizumab + chemotherapy.

In terms of safety during neoadjuvant therapy, we analyzed any grade TRAEs and grade ≥3 TRAEs. The results showed that the incidence rates of any grade TRAEs and grade ≥3 TRAEs were similar across different treatment modalities, with no statistically significant differences. These findings suggest that adding immunotherapy to chemotherapy in the neoadjuvant setting does not significantly increase the risk of severe adverse events. Additionally, we conducted a comprehensive analysis of adverse event outcomes in the included studies to estimate the frequency and severity of certain TRAEs and immune-related adverse events (irAEs). Results indicated that the most common TRAEs in the neoadjuvant chemotherapy plus immunotherapy group were myelosuppression and gastrointestinal damage, primarily including leukopenia (6.7-82.6%), neutropenia (5.3-78.3%), anemia (10.5-100%), thrombocytopenia (0-100%), vomiting (6.7-42.9%), and diarrhea (0-18.8%), with most grade 3 or higher TRAEs being hematologic adverse events. The most common irAEs related to ICIs were rash (4.2-21.7%), thyroid dysfunction (hypothyroidism or hyperthyroidism, 6.3-17.4%), and pneumonitis (4.2-6.3%), predominantly mild to moderate (grade 1 or 2), with no observed grade 4 or higher adverse events. Notably, reactive capillary endothelial proliferation was a specific adverse event associated with Camrelizumab-related skin damage, occurring at a rate of 21.1-60.0%, possibly due to Camrelizumab’s ability to promote capillary proliferation by binding with vascular endothelial growth factor (VEGF). Regarding thyroid dysfunction, the highest risk of adverse events was observed with Pem + nCT (17.4%), highlighting the need for regular thyroid function monitoring in patients receiving Pembrolizumab therapy.

Currently, in clinical trials or studies of neoadjuvant immunotherapy combined with chemotherapy for esophageal cancer, PD-1 inhibitors involved include pembrolizumab, sintilimab, camrelizumab, tislelizumab, toripalimab, and nivolumab. Studies involving toripalimab, tislelizumab, and nivolumab are single-arm studies and relatively limited in number, hence these three PD-1 inhibitors were not included in our study. In addition, some PD-L1 inhibitors combined with chemotherapy have also demonstrated enhanced antitumor activity in treating esophageal cancer. In advanced esophageal cancer, only one phase 2 single-arm study has explored the efficacy and safety of the PD-L1 inhibitor Adebrelimab plus chemotherapy as first-line treatment. In the context of neoadjuvant immunotherapy for locally advanced esophageal cancer, clinical trials primarily focus on exploring the benefits of immunotherapy combined with radiochemotherapy. PD-L1 inhibitors involved in these trials include avelumab, socazolimab, sotigalimab, atezolizumab, and durvalumab, with only one study investigating the benefits of the PD-L1 inhibitor socazolimab combined with chemotherapy. Therefore, large-scale phase 3 RCTs are still needed to study the efficacy of other PD-1 inhibitors and PD-L1 inhibitors in esophageal cancer.

This study also has certain limitations: (1) Currently, there is a lack of clinical trials directly comparing several ICIs, thus preventing validation of indirectly compared results; (2) Studies involving sintilimab + nCT and socazolimab + nCT are relatively limited in number, potentially affecting result reliability; (3) The chemotherapy strategies in this study vary widely, mostly consisting of platinum-based doublet regimens such as cisplatin, nedaplatin, oxaliplatin with paclitaxel, albumin-bound paclitaxel, docetaxel, pemetrexed, and one study with a platinum-based triplet regimen (docetaxel + cisplatin + 5-fluorouracil), suggesting different synergistic effects with immunotherapy; (4) The cases included in this study are all from Chinese populations, possibly introducing regional bias and limiting the generalizability of conclusions to other ethnic groups of esophageal cancer patients; (5) Long-term survival outcome data are lacking in the included studies, a common issue given the considerable time required to obtain such results. However, with the increasing number of active trials in neoadjuvant immunotherapy, future research with larger sample sizes and more RCTs is expected to provide further validation.

## 5 Conclusion

In summary, the existing evidence from this meta-analysis suggests that neoadjuvant immunotherapy combined with chemotherapy regimens demonstrate relatively high efficacy and tolerable safety profiles. Among the perioperative immunotherapy combined chemotherapy regimens evaluated, camrelizumab + nCT showed the highest rates of pCR and MPR, while pembrolizumab + nCT exhibited the highest ORR and DCR. There were no significant differences observed in safety profiles among the groups. Further RCTs are needed, including larger sample sizes, different regional locations, various immunotherapy combined chemotherapy regimens, and longer-term outcomes, to further evaluate the efficacy and safety of these neoadjuvant immunotherapy combined chemotherapy regimens for locally advanced esophageal cancer.

## Data Availability

The original contributions presented in the study are included in the article/**Supplementary Material**. Further inquiries can be directed to the corresponding author.

## References

[1] Bray F Laversanne M Sung H Ferlay J Siegel RL Soerjomataram I . Global cancer statistics 2022: GLOBOCAN estimates of incidence and mortality worldwide for 36 cancers in 185 countries. CA Cancer J Clin. (2024) 74:229–63. doi: 10.3322/caac.21834 38572751

[2] Han BF Zheng RS Zeng HM Wang SM Sun KX Chen R . Cancer incidence and mortality in China, 2022. J Natl Cancer Cent. (2024) 4:47–53. doi: 10.1016/j.jncc.2024.01.006 39036382 PMC11256708

[3] Morgan E Soerjomataram I Rumgay H Coleman HG Thrift AP Vignat J . The global landscape of esophageal squamous cell carcinoma and esophageal adenocarcinoma incidence and mortality in 2020 and projections to 2040: new estimates from GLOBOCAN 2020. Gastroenterology. (2022) 163:649–658.e2. doi: 10.1053/j.gastro.2022.05.054 35671803

[4] Wang LH Yu JM Yu ZT Li Y Wang J Wang GQ . Guideline working committee of chinese society of clinical oncology. In: Chinese Society of Clinical Oncology (CSCO) guidelines for the diagnosis and treatment of esophageal cancer 2022 edition(M). People's Medical Publishing House, Beijing (2022). p. 04.

[5] Watanabe M Otake R Kozuki R Toihata T Takahashi K Okamura A . Recent progress in multidisciplinary treatment for patients with esophageal cancer. Surg Today. (2020) 50:12–20. doi: 10.1007/s00595-019-01878-7 31535225 PMC6952324

[6] Yang H Liu H Chen Y Zhu C Fang W Yu Z . Long-term efficacy of neoadjuvant chemoradiotherapy plus surgery for the treatment of locally advanced esophageal squamous cell carcinoma: the NEOCRTEC5010 randomized clinical trial. JAMA Surg. (2021) 156:721–9. doi: 10.1001/jamasurg.2021.2373 PMC8223138 34160577

[7] Eyck BM van Lanschot JJB Hulshof MCCM van der Wilk BJ Shapiro J van Hagen P . Ten-year outcome of neoadjuvant chemoradiotherapy plus surgery for esophageal cancer: the randomized controlled CROSS trial. J Clin Oncol. (2021) 39:1995–2004. doi: 10.1200/JCO.20.03614 33891478

[8] Kato K Machida R Ito Y Daiko H Ozawa S Ogata T . Doublet chemotherapy, triplet chemotherapy, or doublet chemotherapy combined with radiotherapy as neoadjuvant treatment for locally advanced oesophageal cancer (JCOG1109 NExT): a randomised, controlled, open-label, phase 3 trial. Lancet. (2024) 404:55–66. doi: 10.1016/S0140-6736(24)00745-1 38876133

[9] Ando N Kato H Igaki H Shinoda M Ozawa S Shimizu H . A randomized trial comparing postoperative adjuvant chemotherapy with cisplatin and 5-fluorouracil versus preoperative chemotherapy for localized advanced squamous cell carcinoma of the thoracic esophagus (JCOG9907). Ann Surg Oncol. (2012) 19:68–74. doi: 10.1245/s10434-011-2049-9 21879261

[10] Medina PJ Adams VR . PD-1 pathway inhibitors: immuno-oncology agents for restoring antitumor immune responses. Pharmacotherapy. (2016) 36:317–34. doi: 10.1002/phar.1714 PMC5071694 26822752

[11] Bang YJ Kang YK Catenacci DV Muro K Fuchs CS Geva R . Pembrolizumab alone or in combination with chemotherapy as first-line therapy for patients with advanced gastric or gastroesophageal junction adenocarcinoma: results from the phase II nonrandomized KEYNOTE-059 study. Gastric Cancer. (2019) 22:828–37. doi: 10.1007/s10120-018-00909-5 PMC6570680 30911859

[12] Shah MA Kojima T Hochhauser D Enzinger P Raimbourg J Hollebecque A . Efficacy and safety of pembrolizumab for heavily pretreated patients with advanced, metastatic adenocarcinoma or squamous cell carcinoma of the esophagus: the phase 2 KEYNOTE-180 study. JAMA Oncol. (2019) 5:546–50. doi: 10.1001/jamaoncol.2018 PMC6459121 30570649

[13] Kojima T Shah MA Muro K Francois E Adenis A Hsu CH . Randomized phase III KEYNOTE-181 study of pembrolizumab versus chemotherapy in advanced esophageal cancer. J Clin Oncol. (2020) 38:4138–48. doi: 10.1200/JCO.20.01888 33026938

[14] Janjigian YY Bendell J Calvo E Kim JW Ascierto PA Sharma P . CheckMate-032 study: efficacy and safety of nivolumab and nivolumab plus ipilimumab in patients with metastatic esophagogastric cancer. J Clin Oncol. (2018) 36:2836–44. doi: 10.1200/JCO.2017.76.6212 PMC6161834 30110194

[15] Sun JM Shen L Shah MA Enzinger P Adenis A Doi T . Pembrolizumab plus chemotherapy versus chemotherapy alone for first-line treatment of advanced oesophageal cancer (KEYNOTE-590): a randomised, placebo-controlled, phase 3 study. Lancet. (2021) 398:759–71. doi: 10.1016/S0140-6736(21)01234-4 34454674

[16] Qin H Liu F Zhang Y Liang Y Mi Y Yu F . Comparison of neoadjuvant immunotherapy versus routine neoadjuvant therapy for patients with locally advanced esophageal cancer: A systematic review and meta-analysis. Front Immunol. (2023) 14:1108213. doi: 10.3389/fimmu.2023.1108213 37033991 PMC10076616

[17] Li C Zhao S Zheng Y Han Y Chen X Cheng Z . Preoperative pembrolizumab combined with chemoradiotherapy for oesophageal squamous cell carcinoma (PALACE-1). Eur J Cancer. (2021) 144:232–41. doi: 10.1016/j.ejca.2020.11.039 33373868

[18] van den Ende T de Clercq NC van Berge Henegouwen MI Gisbertz SS Geijsen ED Verhoeven RHA . Neoadjuvant chemoradiotherapy combined with atezolizumab for resectable esophageal adenocarcinoma: A single-arm phase II feasibility trial (PERFECT). Clin Cancer Res. (2021) 27:3351–9. doi: 10.1158/1078-0432.CCR-20-4443 33504550

[19] Peterson J Welch V Losos M Tugwell PJ . The Newcastle-Ottawa scale (NOS) for assessing the quality of non-randomised studies in meta-analyses Vol. 2. . Ottawa: Ottawa Hospital Research Institute (2011) p. 1–2.

[20] Higgins JP Altman DG Gøtzsche PC Jüni P Moher D Oxman AD . The Cochrane Collaboration's tool for assessing risk of bias in randomized trials. BMJ (Clinical Res ed.). (2011) 343:d5928. doi: 10.1136/bmj.d5928 PMC3196245 22008217

[21] Li Y Zhou A Liu S He M Chen K Tian Z . Comparing a PD-L1 inhibitor plus chemotherapy to chemotherapy alone in neoadjuvant therapy for locally advanced ESCC: a randomized Phase II clinical trial: A randomized clinical trial of neoadjuvant therapy for ESCC. BMC Med. (2023) 21:86. doi: 10.1186/s12916-023-02804-y 36882775 PMC9993718

[22] Xiao Y Huo QL Yang YM Lv B Yang XB . Clinical trial of carrelizumab injection combined with operation in the treatment of patients with stage II/III esophageal squamous cell carcinoma. Chin J Clin Pharmacol. (2021) 37:3323–3325+3333. doi: 10.13699/j.cnki.1001-6821.2021.24.005

[23] Qiao Y Zhao C Li X Zhao J Huang Q Ding Z . Efficacy and safety of camrelizumab in combination with neoadjuvant chemotherapy for ESCC and its impact on esophagectomy. Front Immunol. (2022) 13:953229. doi: 10.3389/fimmu.2022.953229 35911723 PMC9329664

[24] Wang S Xu G Li M Zheng J Wang Y Feng X . M1 macrophage predicted efficacy of neoadjuvant camrelizumab combined with chemotherapy vs chemotherapy alone for locally advanced ESCC: A pilot study. Front Oncol. (2023) 13:1139990. doi: 10.3389/fonc.2023.1139990 36969032 PMC10038194

[25] Zhou RQ Luo J Li LJ Du M Wu QC . Neoadjuvant camrelizumab plus chemotherapy in locally advanced oesophageal squamous cell carcinoma: a retrospective cohort study. BMC Surg. (2023) 23:114. doi: 10.1186/s12893-023-02023-5 37161374 PMC10170768

[26] Zhang RQ Song DS Liu W Yao L Chen AG Ge W . Efficacy and safety of camrelizumab combined with chemotherapy versus chemotherapy alone as preoperative neoadjuvant therapy for resectable locally advanced esophageal squamous cell carcinoma: Preliminary results from a multicenter, prospective, randomized controlled study. J Clin Oncol. (2023) 41:abstr 4064. doi: 10.1200/JCO.2023.41.16_suppl.4064

[27] Zhang B Zhao H Wu X Gong L Yang D Li X . Perioperative outcomes of neoadjuvant chemotherapy plus camrelizumab compared with chemotherapy alone and chemoradiotherapy for locally advanced esophageal squamous cell cancer. Front Immunol. (2023) 14:1066527. doi: 10.3389/fimmu.2023.1066527 36825006 PMC9941171

[28] Li XL Jin DY Song YM . Efficacy of camrelizumab combined with albumin - bound paclitaxel and nedaplatin in the treatment of locally advanced esophageal squamous cell carcinoma. Modern Oncol. (2023) 31:4167–71. doi: 10.3969/j.issn.1672-4992.2023.22.013

[29] Chen XF Guo QS . Effect of camrelizumab combined with albumin-binding paclitaxel+cisplatin chemotherapy on preoperative treatment of locally advanced esophageal cancer and its influence on PD-1 and PD-L1 levels. Chin J Mod Drug Appl. (2023) 17:1–5. doi: 10.14164/j.cnki.cn11-5581/r.2023.18.001

[30] Huang B Shi H Gong X Yu J Xiao C Zhou B . Comparison of efficacy and safety between pembrolizumab combined with chemotherapy and simple chemotherapy in neoadjuvant therapy for esophageal squamous cell carcinoma. J Gastrointest Oncol. (2021) 12:2013–21. doi: 10.21037/jgo-21-610 PMC8576253 34790369

[31] Zhang XW Wang RJ Zhang X Lv WD Song YR Lei GY . Clinical efficacy of pembrolizumab combined with neoadjuvant chemotherapy in treatment of stage II and III esophageal cancer. Shaanxi Med J. (2022) 51:870–3. doi: 10.3969/j.issn.1000-7377.2022.07.02

[32] Wang X Liu Z Du Y Zhao B Hao S . Clinical trial of pembrolizumab injection combined with PC regimen in the treatment of patients with esophageal squamous cell carcinoma. Chin J Clin Pharmacol. (2023) 39:936–40. doi: 10.13699/j.cnki.1001-6821.2023.07.006

[33] Chen J Gao YH Ma DD Wu YY Wang YL . Effectiveness of pembrolizumab combined with pemetrexed and cisplatin in preoperative neoadjuvant chemotherapy of esophageal squamous cell carcinoma and its effects on SCCA, CEA, and PD-1 /PD-L1. Med & Pharm J Chin PLA. (2021) 33:23–27+31. doi: 10.3969/j.issn.2095-140X.2021.07.006

[34] Li CL Liu D Gan SY Zhu DQ Li B Wang YL . Efficacy analysis of sintilimab combined with neoadjuvant chemotherapy in treatment of locally advanced esophageal cancer. J Esophageal Dis. (2023) 5:269–74. doi: 10.15926/j.cnki.issn2096-7381.2023.04.006

[35] Liu J Chen Z Li Y Zhao W Wu J Zhang Z . Pd-1/Pd-L1 checkpoint inhibitors in tumor immunotherapy. Front Pharmacol. (2021) 12:731798. doi: 10.3389/fphar.2021.731798 34539412 PMC8440961

[36] Topalian SL Taube JM Pardoll DM . Neoadjuvant checkpoint blockade for cancer immunotherapy. Sci (New York NY). (2020) 367:eaax0182. doi: 10.1126/science.aax0182 PMC7789854 32001626

[37] Wang H Song C Zhao X Deng W Dong J Shen W . Evaluation of neoadjuvant immunotherapy and traditional neoadjuvant therapy for resectable esophageal cancer: a systematic review and single-arm and network meta-analysis. Front Immunol. (2023) 14:1170569. doi: 10.3389/fimmu.2023.1170569 37251393 PMC10213267

[38] He W Wang C Li C Nie X Li H Li J . The efficacy and safety of neoadjuvant immunotherapy in resectable locally advanced esophageal squamous cell carcinoma: A systematic review and meta-analysis. Front Immunol. (2023) 14:1118902. doi: 10.3389/fimmu.2023.1118902 36875107 PMC9981949

[39] Xu J Cai Y Hong Z Duan H Ke S . Comparison of efficacy and safety between neoadjuvant chemotherapy and neoadjuvant immune checkpoint inhibitors combined with chemotherapy for locally advanced esophageal squamous cell carcinoma: a systematic review and meta-analysis. Int J Surg. (2023) 110:490–506. doi: 10.1097/JS9.0000000000000816 PMC10793745 37800587

[40] Mei T Zhou Q Gong Y . Comparison of the efficacy and safety of perioperative immunochemotherapeutic strategies for resectable non-small cell lung cancer: a systematic review and network meta-analysis. Clin Oncol (R Coll Radiol). (2023) 36:107–18. doi: 10.1016/j.clon.2023.12.006 38151439

[41] Li ZC Sun YT Lai MY Zhou YX Qiu MZ . Efficacy and safety of PD-1 inhibitors combined with chemotherapy as first-line therapy for advanced esophageal cancer: A systematic review and network meta-analysis. Int Immunopharmacol. (2022) 109:108790. doi: 10.1016/j.intimp.2022.108790 35504202

[42] Gao TT Shan JH Yang YX Zhang ZW Liu SL Xi M . Comparative efficacy and safety of immunotherapy for patients with advanced or metastatic esophageal squamous cell carcinoma: a systematic review and network Meta-analysis. BMC Cancer. (2022) 22:992. doi: 10.1186/s12885-022-10086-5 36115960 PMC9482734

