# Supplementary material for: Comparison of the efficacy and safety of perioperative immunochemotherapeutic strategies for locally advanced esophageal cancer: a systematic review and network meta-analysis
Source: Front Immunol. 2024 Dec 6;15:1478377. doi: 10.3389/fimmu.2024.1478377 (PMC11659204; doi:10.3389/fimmu.2024.1478377)

Supplementary Material

[Supplementary Table 1 Search strategy 2](#_Toc78268073)

[Supplementary File 1](#_Toc78268074)[The PRISMA checklist of this meta-analysis 4](#_Toc78268074)

[Supplementary Table 2 Risk of bias evaluation of the included RCTs 7](#_Toc78268075)

[Supplementary Table 3 Quality assessment of included studies based on the Newcastle-Ottawa Scale score. 8](#_Toc78268075)

[Supplementary Figure 1 Network evidence Plot 9](#_Toc78268075)

[Supplementary Figure 2 Predictive intervals plots for safety 1](#_Toc78268075)0

[Supplementary Figure 3 Cumulative ranking probability graphs for safety 1](#_Toc78268075)1

# Supplementary Table 1 Search strategy

| **Databases** | **Search items** |
| --- | --- |
| PubMed | ((((((((((((((((((((((((((((immunotherapy[Title/Abstract]) OR ("immune checkpoint inhibitors"[Title/Abstract]) OR ("ICI"[Title/Abstract])) OR ("programmed cell death 1 receptor"[Title/Abstract])) OR ("programmed cell death ligand 1"[Title/Abstract])) OR ("cytotoxic T lymphocyte-associated antigen-4 antigen"[Title/Abstract])) OR ("CTLA-4 antigen"[Title/Abstract])) OR (anti-CTLA-4[Title/Abstract])) OR (CTLA-4[Title/Abstract])) OR (anti-PD-1[Title/Abstract])) OR (anti-PD-L1[Title/Abstract])) OR (PD-1[Title/Abstract])) OR (PD-L1[Title/Abstract])) OR (Avelumab[Title/Abstract])) OR (Atezolizumab[Title/Abstract])) OR (Cemiplimab[Title/Abstract])) OR (Camrelizumab[Title/Abstract])) OR (Durvalumab[Title/Abstract])) OR (Ipilimumab[Title/Abstract])) OR (Lambrolizumab[Title/Abstract])) OR (Nivolumab[Title/Abstract])) OR (Pembrolizumab[Title/Abstract])) OR (Sintilimab[Title/Abstract])) OR (Tremelimumab[Title/Abstract])) OR (Tislelizumab[Title/Abstract])) OR (Toripalimab[Title/Abstract])) ) AND ((((Neoadjuvant[Title/Abstract]) OR (Preoperative[Title/Abstract])) OR (Perioperative[Title/Abstract]))) AND ((((((((((((((((((("Esophageal Neoplasms"[MeSH Terms]) OR ("Cancer of Esophagus"[Title/Abstract])) OR ("Cancer of the Esophagus"[Title/Abstract])) OR ("Esophageal Cancer"[Title/Abstract])) OR ("Esophagus Cancer"[Title/Abstract])) OR ("esophageal neoplasm"[Title/Abstract])) OR ("Esophagus Neoplasm"[Title/Abstract])) OR ("carcinoma of esophagus"[Title/Abstract])) OR ("Esophageal squamous cell carcinoma"[Title/Abstract])) OR ("Esophageal squamous carcinoma"[Title/Abstract])) OR ("Esophageal squamous cell cancer"[Title/Abstract])) OR ("Esophageal carcinoma"[Title/Abstract])) OR ("esophageal adenocarcinoma"[Title/Abstract])) OR ("esophageal malignancy"[Title/Abstract])) OR (ESCC[Title/Abstract])) OR (Esophagus[Title/Abstract])) OR (Esophageal[Title/Abstract])) OR (oesophagus[Title/Abstract])) OR (oesophageal[Title/Abstract])) |
| EMBASE | (immunotherapy:ti,ab,kw OR 'immune checkpoint inhibitors':ti,ab,kw OR 'ICI':ti,ab,kw OR 'programmed cell death 1 receptor':ti,ab,kw OR 'programmed cell death ligand 1':ti,ab,kw OR 'cytotoxic t lymphocyte-associated antigen-4 antigen':ti,ab,kw OR 'ctla-4 antigen':ti,ab,kw OR 'anti ctla 4':ti,ab,kw OR 'ctla 4':ti,ab,kw OR 'anti pd 1':ti,ab,kw OR 'anti pd l1':ti,ab,kw OR 'pd 1':ti,ab,kw OR 'pd l1':ti,ab,kw OR atezolizumab:ti,ab,kw OR avelumab:ti,ab,kw OR cemiplimab:ti,ab,kw OR camrelizumab:ti,ab,kw OR durvalumab:ti,ab,kw OR ipilimumab:ti,ab,kw OR lambrolizumab:ti,ab,kw OR nivolumab:ti,ab,kw OR pembrolizumab:ti,ab,kw OR sintilimab:ti,ab,kw OR toripalimab:ti,ab,kw OR tremelimumab:ti,ab,kw OR tislelizumab:ti,ab,kw) AND (neoadjuvant:ti,ab,kw OR preoperative:ti,ab,kw OR perioperative:ti,ab,kw) AND ('esophageal neoplasms':ti,ab,kw OR 'cancer of esophagus':ti,ab,kw OR 'cancer of the esophagus':ti,ab,kw OR 'esophageal cancer':ti,ab,kw OR 'esophagus cancer':ti,ab,kw OR 'esophageal neoplasm':ti,ab,kw OR 'esophagus neoplasm':ti,ab,kw OR 'carcinoma of esophagus':ti,ab,kw OR 'esophageal squamous cell carcinoma':ti,ab,kw OR 'esophageal squamous carcinoma':ti,ab,kw OR 'esophageal squamous cell cancer':ti,ab,kw OR 'esophageal carcinoma':ti,ab,kw OR 'esophageal adenocarcinoma':ti,ab,kw OR 'esophageal malignancy':ti,ab,kw OR escc:ti,ab,kw OR esophagus:ti,ab,kw OR esophageal:ti,ab,kw OR oesophagus:ti,ab,kw OR oesophageal:ti,ab,kw) |
| Cochrane Library | ((immunotherapy):ti,ab,kw OR (“immune checkpoint inhibitors”):ti,ab,kw OR (ICI):ti,ab,kw OR (“programmed cell death 1 receptor”):ti,ab,kw OR (“programmed cell death ligand 1”):ti,ab,kw OR (“cytotoxic T lymphocyte-associated antigen-4 antigen”):ti,ab,kw OR (“CTLA-4 antigen”):ti,ab,kw OR (anti-CTLA-4):ti,ab,kw OR (CTLA-4):ti,ab,kw OR (anti-PD-1):ti,ab,kw OR (anti-PD-L1):ti,ab,kw OR (PD-1):ti,ab,kw OR (PD-L1):ti,ab,kw OR (Avelumab):ti,ab,kw OR (Atezolizumab):ti,ab,kw OR (Cemiplimab):ti,ab,kw OR (Camrelizumab):ti,ab,kw OR (Durvalumab):ti,ab,kw OR (Ipilimumab):ti,ab,kw OR (Lambrolizumab):ti,ab,kw OR (Nivolumab):ti,ab,kw OR (Pembrolizumab):ti,ab,kw OR (Sintilimab):ti,ab,kw OR (Toripalimab):ti,ab,kw OR (Tislelizumab):ti,ab,kw OR (Tremelimumab):ti,ab,kw) AND ((Neoadjuvant):ti,ab,kw OR (Preoperative):ti,ab,kw OR (Perioperative):ti,ab,kw) AND (("Esophageal Neoplasms"):ti,ab,kw OR ("Cancer of Esophagus"):ti,ab,kw OR ("Cancer of the Esophagus"):ti,ab,kw OR ("Esophageal Cancer"):ti,ab,kw OR ("Esophagus Cancer"):ti,ab,kw OR (“esophageal neoplasm”):ti,ab,kw OR ("Esophagus Neoplasm"):ti,ab,kw OR ("carcinoma of esophagus"):ti,ab,kw OR ("Esophageal squamous cell carcinoma"):ti,ab,kw OR ("Esophageal squamous carcinoma"):ti,ab,kw OR (“Esophageal squamous cell cancer”):ti,ab,kw OR ("Esophageal carcinoma"):ti,ab,kw OR (“esophageal adenocarcinoma”):ti,ab,kw OR (“esophageal malignancy”):ti,ab,kw OR (ESCC):ti,ab,kw OR (Esophagus):ti,ab,kw OR (Esophageal):ti,ab,kw OR (oesophagus):ti,ab,kw OR (oesophageal):ti,ab,kw) |
| Web of Science | (((((((((((((((((((((((((((TS=(immunotherapy)) OR TS=(“immune checkpoint inhibitors”) OR TS=(“ICI”)) OR TS=(“programmed cell death 1 receptor”)) OR TS=(“programmed cell death ligand 1”)) OR TS=(“cytotoxic T lymphocyte-associated antigen-4 antigen”)) OR TS=(“CTLA-4 antigen”)) OR TS=(anti-CTLA-4)) OR TS=(CTLA-4)) OR TS=(anti-PD-1)) OR TS=(anti-PD-L1)) OR TS=(PD-1)) OR TS=(PD-L1)) OR TS=(Durvalumab)) OR TS=(Atezolizumab)) OR TS=(Avelumab)) OR TS=(Camrelizumab)) OR TS=(cemiplimab)) OR TS=(Ipilimumab)) OR TS=(Lambrolizumab)) OR TS=(Nivolumab)) OR TS=(Pembrolizumab)) OR TS=(Sintilimab)) OR TS=(Tremelimumab)) OR TS=(Tislelizumab)) OR TS=(Toripalimab)))) AND ((((TS=(Neoadjuvant)) OR TS=(Preoperative)) OR TS=(Perioperative)) AND (((((((((((((((((((TS=("Esophageal Neoplasms")) OR TS=("Cancer of Esophagus")) OR TS=("Cancer of the Esophagus")) OR TS=("Esophageal Cancer")) OR TS=("Esophagus Cancer")) OR TS=(“esophageal neoplasm”)) OR TS=("Esophagus Neoplasm")) OR TS=("carcinoma of esophagus")) OR TS=("Esophageal squamous cell carcinoma")) OR TS=("Esophageal squamous carcinoma")) OR TS=(“Esophageal squamous cell cancer”)) OR TS=("Esophageal carcinoma")) OR TS=(“esophageal adenocarcinoma”)) OR TS=(“esophageal malignancy”)) OR TS=(ESCC)) OR TS=(Esophagus)) OR TS=(Esophageal)) OR TS=(oesophagus)) OR TS=(oesophageal)) |
| CNKI | TKA=(免疫 + 度伐利尤单抗 + 阿替利珠单抗 + 阿维单抗 + 帕博利珠单抗 + 纳武利尤单抗 + 特瑞普利单抗 + 替雷利珠单抗 + 卡瑞丽珠单抗 + 信迪力单抗 + 替西木单抗 + 伊匹单抗 + 派姆单抗 + 西米普利单抗) * (新辅助 + 围术期 + 术前) * (食管癌 + 食管鳞状细胞癌 + 食管腺癌) |
| Wanfang | 主题:(免疫 OR 度伐利尤单抗 OR 阿替利珠单抗 OR 阿维单抗 OR 帕博利珠单抗 OR 纳武利尤单抗 OR 特瑞普利单抗 OR 替雷利珠单抗 OR 卡瑞丽珠单抗 OR 信迪力单抗 OR 替西木单抗 OR 伊匹单抗 OR 派姆单抗 OR 西米普利单抗) AND (新辅助 OR 围术期 OR 术前) AND (食管癌 OR 食管鳞状细胞癌 OR 食管腺癌) |

**Supplementary File 1 The PRISMA checklist of this meta-analysis**

| **Section/topic** | **#** | **Checklist item** | **Reported on page #** |
| --- | --- | --- | --- |
| **TITLE** | | |  |
| Title | 1 | Identify the report as a systematic review, meta-analysis, or both. | 1 |
| **ABSTRACT** | | |  |
| Structured summary | 2 | Provide a structured summary including, as applicable: background; objectives; data sources; study eligibility criteria, participants, and interventions; study appraisal and synthesis methods; results; limitations; conclusions and implications of key findings; systematic review registration number. | 1-2 |
| **INTRODUCTION** | | |  |
| Rationale | 3 | Describe the rationale for the review in the context of what is already known. | 2 |
| Objectives | 4 | Provide an explicit statement of questions being addressed with reference to participants, interventions, comparisons, outcomes, and study design (PICOS). | 2 |
| **METHODS** | | |  |
| Protocol and registration | 5 | Indicate if a review protocol exists, if and where it can be accessed (e.g., Web address), and, if available, provide registration information including registration number. | no |
| Eligibility criteria | 6 | Specify study characteristics (e.g., PICOS, length of follow-up) and report characteristics (e.g., years considered, language, publication status) used as criteria for eligibility, giving rationale. | 3 |
| Information sources | 7 | Describe all information sources (e.g., databases with dates of coverage, contact with study authors to identify additional studies) in the search and date last searched. | 3 |
| Search | 8 | Present full electronic search strategy for at least one database, including any limits used, such that it could be repeated. | 3; Supplementary Table 1 |
| Study selection | 9 | State the process for selecting studies (i.e., screening, eligibility, included in systematic review, and, if applicable, included in the meta-analysis). | 3 |
| Data collection process | 10 | Describe method of data extraction from reports (e.g., piloted forms, independently, in duplicate) and any processes for obtaining and confirming data from investigators. | 4 |
| Data items | 11 | List and define all variables for which data were sought (e.g., PICOS, funding sources) and any assumptions and simplifications made. | 4 |
| Risk of bias in individual studies | 12 | Describe methods used for assessing risk of bias of individual studies (including specification of whether this was done at the study or outcome level), and how this information is to be used in any data synthesis. | 4 |
| Summary measures | 13 | State the principal summary measures (e.g., risk ratio, difference in means). | 4 |
| Synthesis of results | 14 | Describe the methods of handling data and combining results of studies, if done, including measures of consistency (e.g., I^2^) for each meta-analysis. | 4 |

| **Section/topic** | **#** | **Checklist item** | **Reported on page #** |
| --- | --- | --- | --- |
| Risk of bias across studies | 15 | Specify any assessment of risk of bias that may affect the cumulative evidence (e.g., publication bias, selective reporting within studies). | no |
| Additional analyses | 16 | Describe methods of additional analyses (e.g., sensitivity or subgroup analyses, meta-regression), if done, indicating which were pre-specified. | no |
| **RESULTS** | | |  |
| Study selection | 17 | Give numbers of studies screened, assessed for eligibility, and included in the review, with reasons for exclusions at each stage, ideally with a flow diagram. | 4; Figure1 |
| Study characteristics | 18 | For each study, present characteristics for which data were extracted (e.g., study size, PICOS, follow-up period) and provide the citations. | 4-5; Table1-2 |
| Risk of bias within studies | 19 | Present data on risk of bias of each study and, if available, any outcome level assessment (see item 12). | 5;Table 3-4 |
| Results of individual studies | 20 | For all outcomes considered (benefits or harms), present, for each study: (a) simple summary data for each intervention group (b) effect estimates and confidence intervals, ideally with a forest plot. | 5-6;  Figure 3-4 |
| Synthesis of results | 21 | Present results of each meta-analysis done, including confidence intervals and measures of consistency. | 5-6 |
| Risk of bias across studies | 22 | Present results of any assessment of risk of bias across studies (see Item 15). | no |
| Additional analysis | 23 | Give results of additional analyses, if done (e.g., sensitivity or subgroup analyses, meta-regression [see Item 16]). | no |
| **DISCUSSION** | | |  |
| Summary of evidence | 24 | Summarize the main findings including the strength of evidence for each main outcome; consider their relevance to key groups (e.g., healthcare providers, users, and policy makers). | 6-8 |
| Limitations | 25 | Discuss limitations at study and outcome level (e.g., risk of bias), and at review-level (e.g., incomplete retrieval of identified research, reporting bias). | 8 |
| Conclusions | 26 | Provide a general interpretation of the results in the context of other evidence, and implications for future research. | 8 |
| **FUNDING** | | |  |
| Funding | 27 | Describe sources of funding for the systematic review and other support (e.g., supply of data); role of funders for the systematic review. | 9 |

*From:*  Moher D, Liberati A, Tetzlaff J, Altman DG, The PRISMA Group (2009). Preferred Reporting Items for Systematic Reviews and Meta-Analyses: The PRISMA Statement. PLoS Med 6(6): e1000097. doi:10.1371/journal.pmed1000097

For more information, visit: **[www.prisma-statement.org.](http://www.prisma-statement.org.)**

**Supplementary Table 2** Risk of bias evaluation of the included RCTs.

| **Study** | **Random sequence generation** | **Allocation Concealment** | **Blinding**  **in performance** | **Blinding of**  **outcome assessment** | **Incomplete outcome data** | **Selective reporting** | **Other bias** | **Total** |
| --- | --- | --- | --- | --- | --- | --- | --- | --- |
| Yong Li 2023 | Low | Low | Low | Low | Low | Low | Low | Low |
| Yong Xiao 2021 | Low | Unclear | High | High | low | Low | Low | High |
| Shu Wang 2023 | Low | Unclear | Unclear | Unclear | Low | Low | Unclear | SC |
| Renquan Zhang 2023 | Low | Unclear | Low | Low | Low | Low | Unclear | SC |
| Xianfang Chen 2023 | Low | Unclear | Unclear | Low | Low | Low | Unclear | SC |
| Xinwei Zhang 2022 | Low | Unclear | Unclear | Unclear | Low | Low | Unclear | SC |

*SC= some concerns*

**Supplementary Table 3** Quality assessment of included studies based on the Newcastle-Ottawa Scale score.

| **Study** | **Representativeness of the exposed cohort** | **Selection of the non-exposed cohort** | **Ascertainment of exposure** | **Demonstration that outcome of interest was not present at start of study** | **Comparability of cohorts on the basis of the design or analysis** | **Assessment of outcome** | **Was follow-up long enough for outcomes to occur** | **Adequacy of follow up of cohorts** | **Total score** |
| --- | --- | --- | --- | --- | --- | --- | --- | --- | --- |
| Yujin Qiao 2022 | 🟓 | 🟓 | 🟓 | 🟓 | 🟓🟓 | 🟓 | 🟓 | 🟓 | 9 |
| Ruiqin Zhou 2023 | 🟓 | 🟓 | 🟓 | 🟓 | 🟓 | 🟓 | 🟓 | 🟓 | 8 |
| Baihua Zhang 2023 | 🟓 | 🟓 | 🟓 | 🟓 | 🟓🟓 | 🟓 | - | 🟓 | 8 |
| Xiaolin Li 2023 | 🟓 | 🟓 | 🟓 | 🟓 | 🟓 | 🟓 | - | 🟓 | 7 |
| Bingjiang Huang 2021 | 🟓 | 🟓 | 🟓 | 🟓 | 🟓🟓 | 🟓 | - | 🟓 | 8 |
| Chunlin Li 2023 | 🟓 | 🟓 | 🟓 | 🟓 | 🟓 | 🟓 | - | 🟓 | 7 |
| Xuezhong Wang 2023 | 🟓 | 🟓 | 🟓 | 🟓 | 🟓 | 🟓 | - | 🟓 | 7 |
| Jing Chen 2021 | 🟓 | 🟓 | 🟓 | 🟓 | 🟓 | 🟓 | - | 🟓 | 7 |

**Supplementary Figure 1** Network Evidence Plot of (A) Major Pathological Response (MPR), (B) R0 resection rate, (C) Objective response rate (ORR), (D) Disease control rate (DCR), (E) Any-grade TRAEs, (F) Grade≥3 TRAEs.


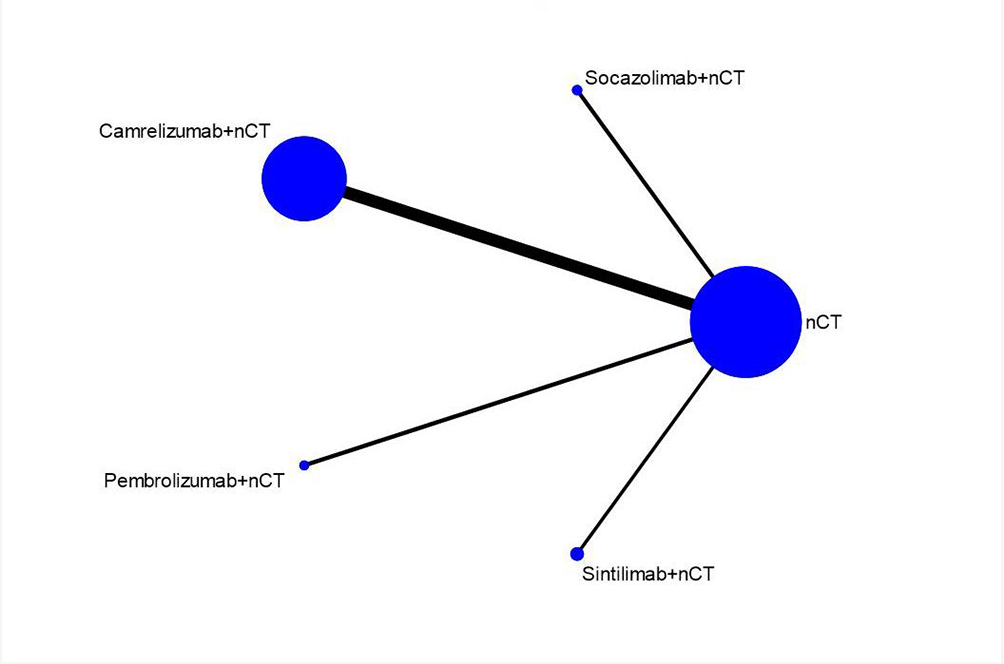

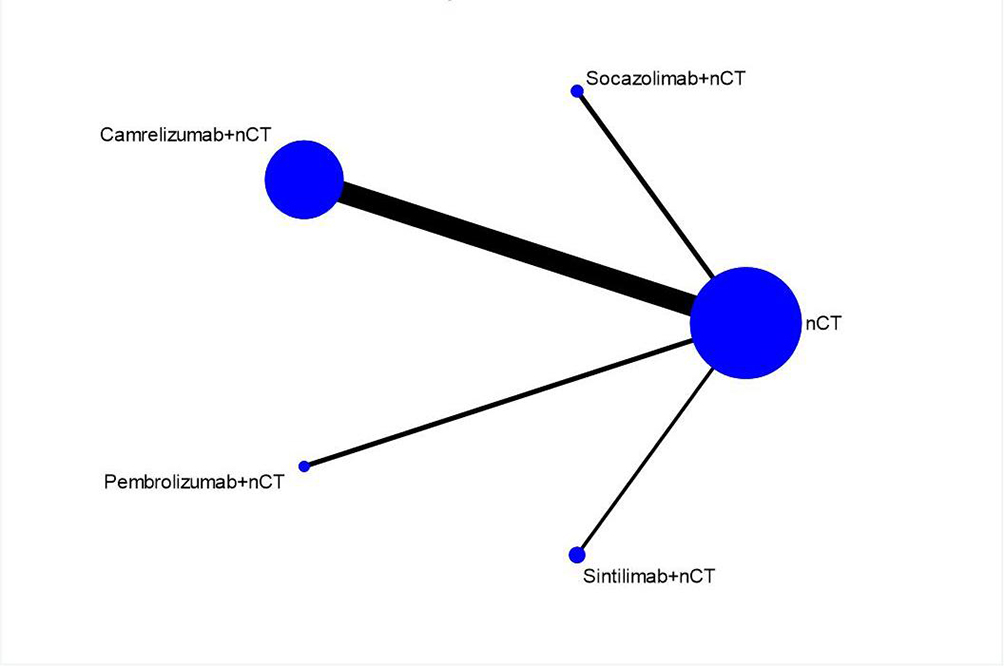


（A）

（B）


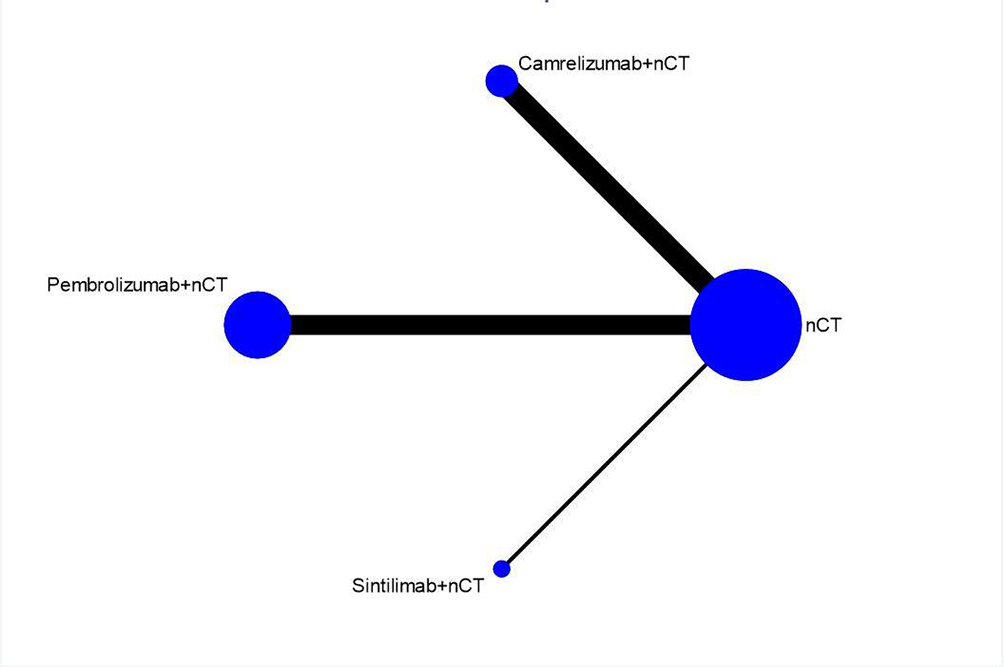

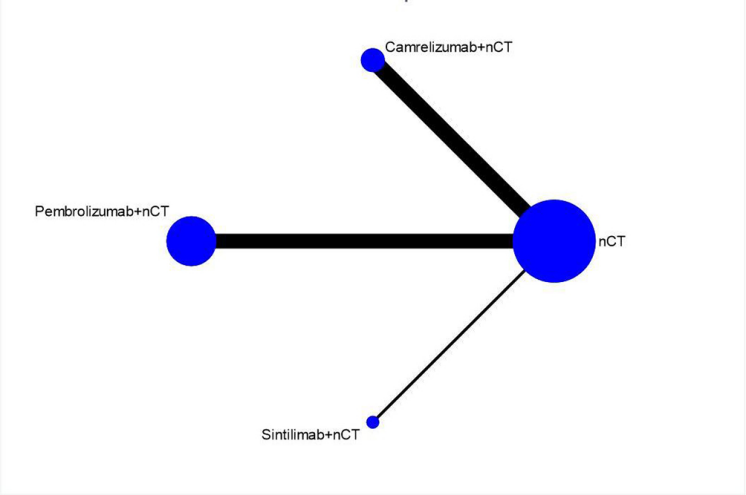


（C）

（D）


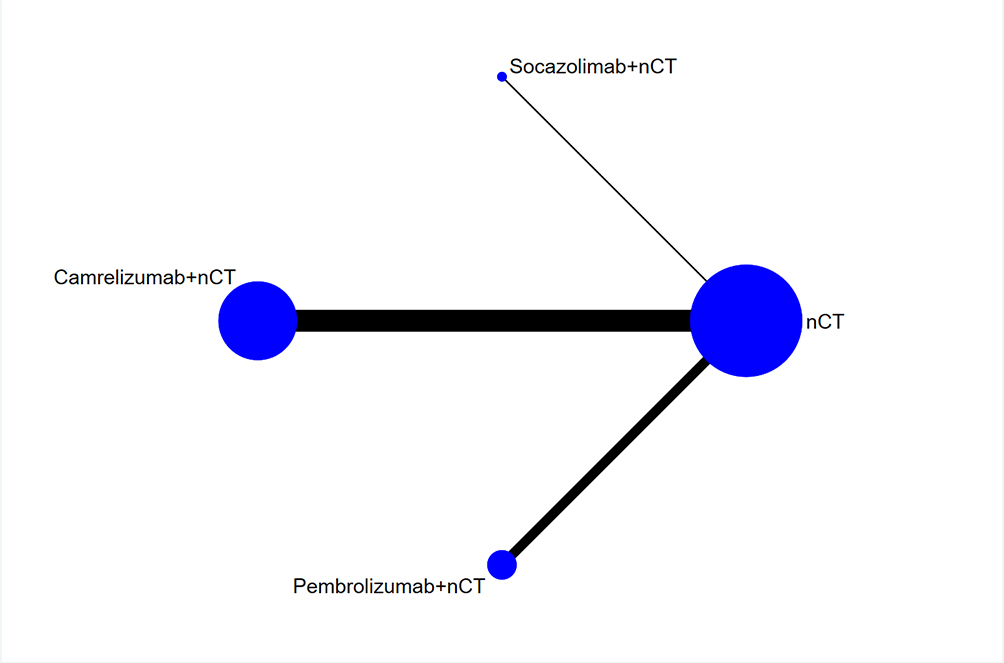

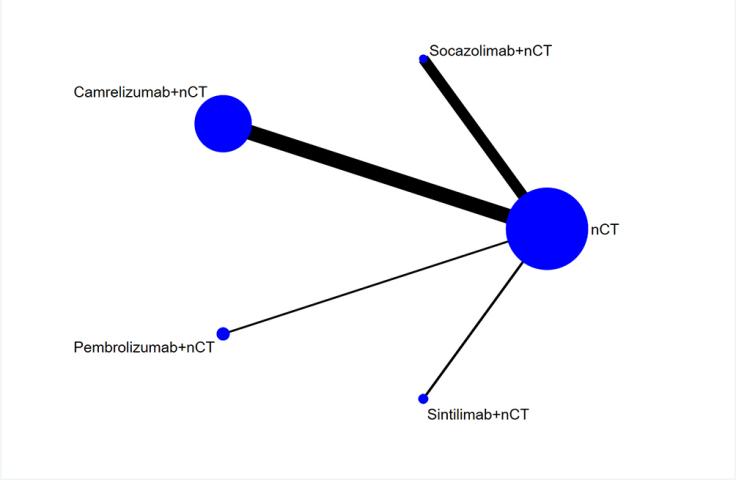


（E）

（F）

**Supplementary Figure 2.** Predictive intervals plots for safety of immunochemotherapeutic strategies. (A) Any-grade TRAEs, (B) Grade≥3 TRAEs.

（A）

（B）


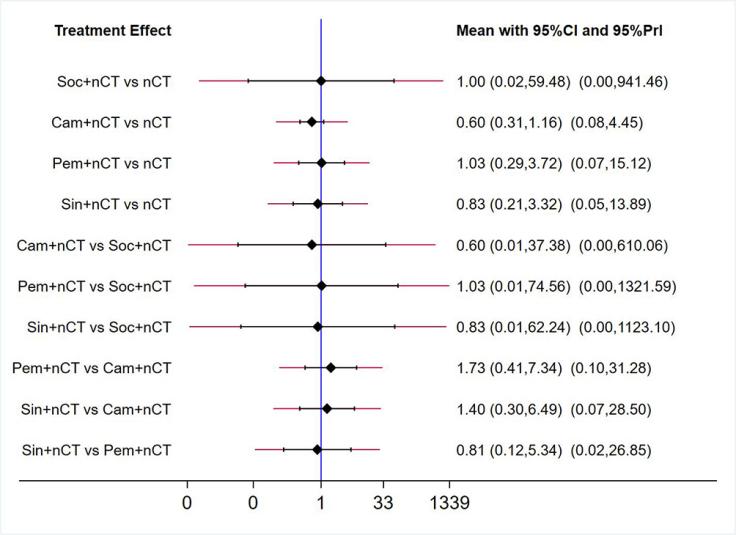

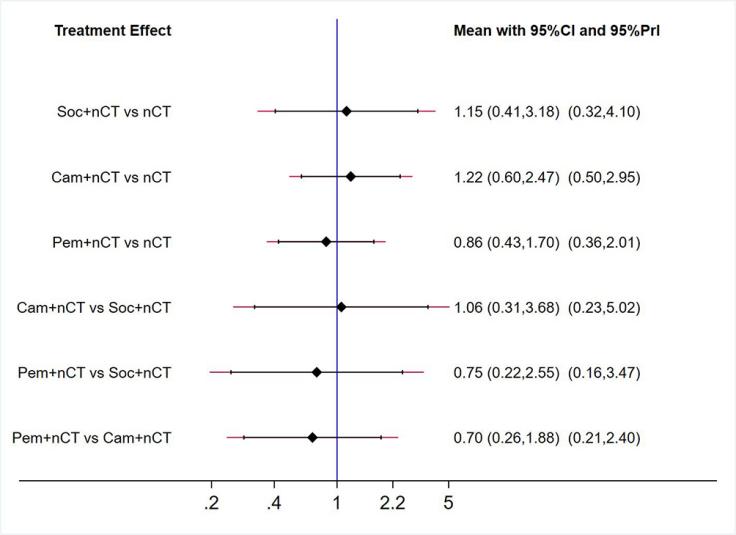


**Supplementary Figure 3.** Cumulative ranking probability graphs for safety of immunochemotherapeutic strategies. (A) Any-grade TRAEs, (B) Grade≥3 TRAEs.

（A）

（B）


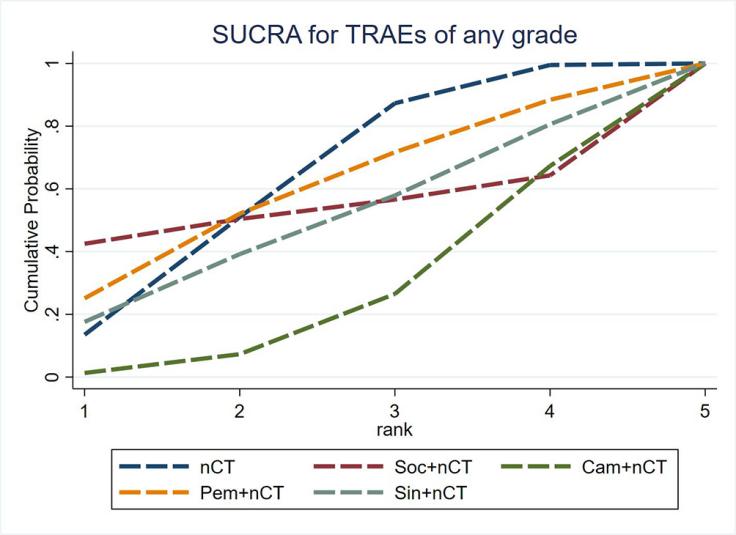

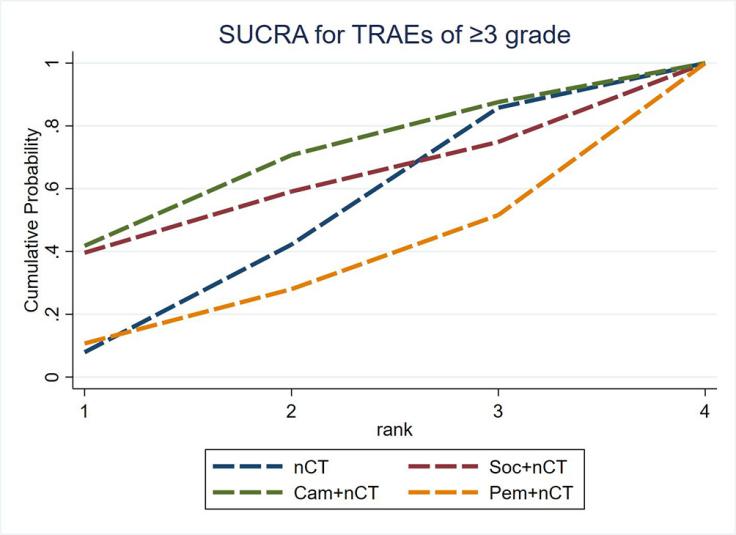

Supplement: Supplementary file 1 [file DataSheet1.docx]
